# A New MEMS Gyroscope Used for Single-Channel Damping

**DOI:** 10.3390/s150510146

**Published:** 2015-04-30

**Authors:** Zengping Zhang, Wei Zhang, Fuxue Zhang, Biao Wang

**Affiliations:** 1 School of Computer and Information Management, Inner Mongolia University of Finance and Economics, Hohhot 010070, China; E-Mail: wangbiao126com@126.com; 2 Sensing Technique Research Center, Beijing Information Science and Technology University, Beijing 100101, China; E-Mails: way_zh@163.com (W.Z.); zhangfuxue@263.net (F.Z.)

**Keywords:** MEMS gyroscope, sensing element, motion equation, steady state solution

## Abstract

The silicon micromechanical gyroscope, which will be introduced in this paper, represents a novel MEMS gyroscope concept. It is used for the damping of a single-channel control system of rotating aircraft. It differs from common MEMS gyroscopes in that does not have a drive structure, itself, and only has a sense structure. It is installed on a rotating aircraft, and utilizes the aircraft spin to make its sensing element obtain angular momentum. When the aircraft is subjected to an angular rotation, a periodic Coriolis force is induced in the direction orthogonal to both the angular momentum and the angular velocity input axis. This novel MEMS gyroscope can thus sense angular velocity inputs. The output sensing signal is exactly an amplitude-modulation signal. Its envelope is proportional to the input angular velocity, and the carrier frequency corresponds to the spin frequency of the rotating aircraft, so the MEMS gyroscope can not only sense the transverse angular rotation of an aircraft, but also automatically change the carrier frequency over the change of spin frequency, making it very suitable for the damping of a single-channel control system of a rotating aircraft. In this paper, the motion equation of the MEMS gyroscope has been derived. Then, an analysis has been carried to solve the motion equation and dynamic parameters. Finally, an experimental validation has been done based on a precision three axis rate table. The correlation coefficients between the tested data and the theoretical values are 0.9969, 0.9872 and 0.9842, respectively. These results demonstrate that both the design and sensing mechanism are correct.

## 1. Introduction

For rotating aircraft, the attitude motion usually has three degrees-of-freedom (also called three channels) including pitch, yaw and rolling. According to the channel selection, the control systems of an aircraft can be divided into single-channel control systems and multi-channel control systems. For some aircraft, which rotate around their longitudinal axis at high rate, a single-channel control system is a good choice [1,2,3], with many benefits such as less equipment, simple structure, light mass and a space saving layout. In the stable loop of a single-channel control system, the angular rate gyroscope is the key device. It is responsible for measuring the pitch and yaw of the rotating aircraft body, then it feeds the output signal back to the input end of the control system so that control system can adjust flight accuracy in time.

With the development of MEMS technology, MEMS gyroscopes are widely used in the areas of inertial navigation and guidance, since they many advantages including low cost, light mass, small volume and anti-shock properties, *etc.* [4,5,6]. Most MEMS gyroscopes are basically a vibratory gyroscope based on Coriolis force [7,8,9,10].

Common MEMS gyroscopes not only have sense structures, but also drive structures. In order to attain the maximum possible response gain and sensitivity, it is necessary to utilize resonance in both the drive and sense modes. This is mainly achieved by structure design or tuning the drive structure and sense structure resonant frequency to match [11]. This paper will introduce a novel MEMS gyroscope, which has no drive structure, and only a sense structure [12,13]. It is mounted on a rotating aircraft, and can smartly determine the angular momentum by using the spin of the rotating aircraft, so it does not need a drive structure. Its proof-mass only has the detecting mode and its structure design is relatively simple, so its fabrication process is also not complex.

In addition, because of the high rate spin of rotating aircraft, a Magnus effect will exist. Generally, when we need to measure a transverse angular velocity input, two common MEMS gyroscopes will be orthogonally mounted on the rotating aircraft, and both the pitch channel and the yaw channel would produce coupling [14]. In applications, it is found that the measured precision will be reduced and some form of compensation is usually necessary for common gyroscopes.

The non-driven MEMS gyroscope, which will be introduced in this paper, is installed on the rotating aircraft. When the aircraft is subjected to a transverse rotation, it can directly sense the transverse angular velocity input. In other words, it senses the resultant angular velocity of pitch and yaw. The amplitude of the output signal is proportional to the transverse angular velocity input. Then the output signal will be directly fed back to the input end of the control system, so the non-driven MEMS gyroscope does not need to compensate for the influence of the Magnus effect. What is more, it can automatically change the carrier frequency of the output signal with the change of spin frequency of the rotating aircraft, making it very suitable for damping in the stable loop of a single-channel control system for a rotating aircraft.

## 2. The Sensing Mechanism

**Figure 1:**
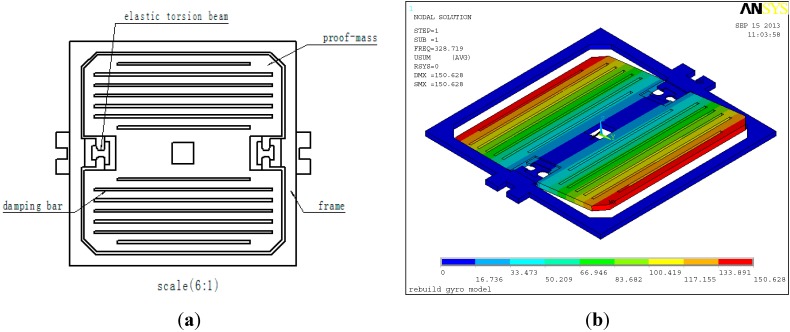
(**a**) The structural diagram; (**b**) the vibrating mode.

The sensing element of the MEMS gyroscope is fabricated using a bulk micromechanical process on a single-crystalline silicon wafer. Figure 1 is the structural diagram and the vibrating mode of the sensing element.

Figure 1a shows that the sensing element is mainly composed of an outer frame and proof-mass. Two ends of the mass are elastic torsion beams. The proof-mass is suspended above the outer frame by a suspension system consisting of flexible beams. The outer frame is fixed on the substrate. The sensing element only has one degree-of-freedom, around which the proof-mass can produce vibrations. This DOF is the constrained center line formed by the elastic torsion beams, so the sensing element only has a sense structure, and no drive structure.

Figure 1b is the first order vibrational mode obtained by finite element analysis (FEA). In modal FEA analysis, according to the size of the sensing element and the material constants of single-crystal silicon, the type of sensing element is defined as SOLID45. Meanwhile, the analyst chooses SI units and sets EX=130.2 GP_a_, PRXY=0.279 and $DENS=2.34\;\times \;10^{3}$ kg/m^3^. The modal analysis result is shown in Figure 1b. Max displacement is about $1.51\;\times \;10^{-11}$ m, and the natural frequency is about 329 Hz.

**Figure 2:**
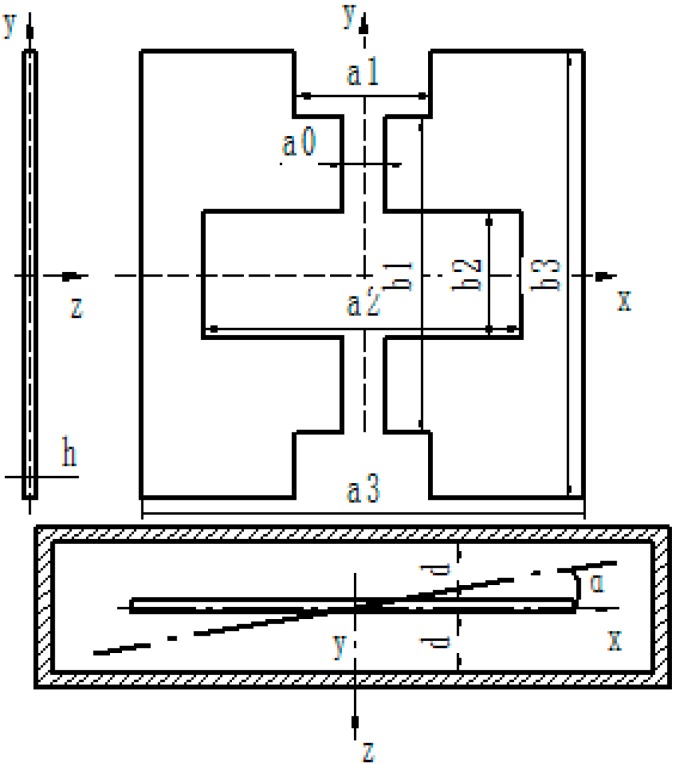
The packaging schematic.

In most of the reported MEMS vibratory rate gyroscopes, the proof-mass is driven into resonance in the drive direction by an external sinusoidal electrostatic or electromagnetic force. Ideally, it is desired to utilize resonance in both the drive and the sense modes. This is typically achieved by designing and if needed tuning the drive and sense resonant frequencies to match. Alternatively, the sense-mode is designed to be slightly shifted from the drive-mode to improve robustness and thermal stability, while intentionally sacrificing gain and sensitivity [11]. The MEMS gyroscope introduced in this paper is a non-driven gyro. The proof-mass does not utilize any drive force such as an electrostatic or electromagnetic force to attain angular momentum. Here, the sensing element is packaged under a nitrogen environment, but is not vacuum packaged. As shown in Figure 2, the use of a squeezed film to produce a damping moment is desired.

The packaging uses the parallel seam welding process, and impermeability is checked using the Bombing method. In this method, the tested device will be placed in a container, and then the container is filled with a certain pressure of helium. After 5–6 h, the tested device is taken out, and then placed in another container. The container is linked to a helium mass spectrometer leak detector. Finally, the impermeability of MEMS gyro can be measured by the leak detector.

In addition, because of the lack of a drive structure, the non-driven MEMS gyro does not need to tune the driving and sensing resonant frequencies to match to attain high gain and sensitivity, so its design is relatively simple, and the fabrication process is also not complex.

**Figure 3:**
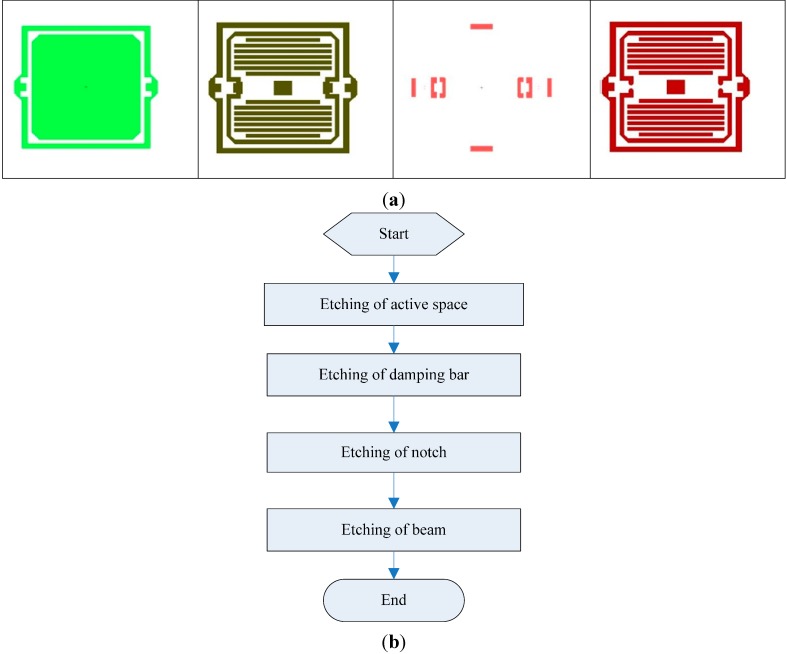
(**a**) The step of photo-mask; (**b**) The fabrication flow of the sensing element.

Twenty-five sensing elements can be fabricated on a single wafer, including oxidation, patterned by lithography, etching and so on. Figure 3a shows four lithography photo-masks. Figure 3b shows the fabrication flow of the sensing element. It includes four steps. In the first step, on a cleaned silicon wafer, oxidation will grow a 700 nm thick SiO_2_ layer. Then through lithography and etching, the active space of sensing element will be obtained. In the second step the damping hole is etched, and then the thickness of the beam is reserved. In the third step the notch of the beam is etched. Finally, in the fourth step the buffer layer of the beam is etched, and beam structure will be formed. After the above four steps, the sensing element is finished.

**Figure 4:**
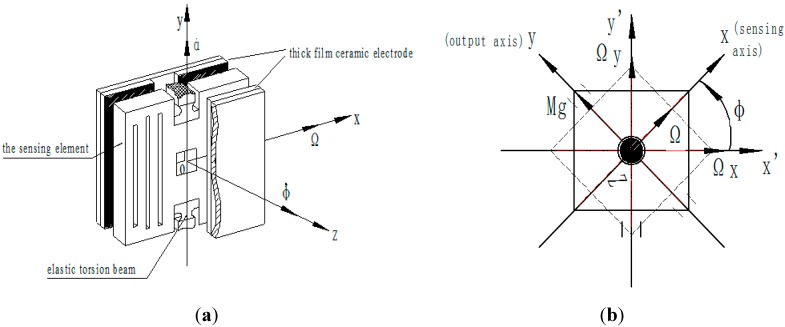
(**a**) The structural diagram of the gyro; (**b**) The diagram of the working principle.

This new MEMS gyroscope is installed on a rotating aircraft. The sensing element of the gyroscope will smartly utilize the spin of the rotating aircraft to drive the proof-mass and obtain angular momentum. When the aircraft is subject to a transverse rotation, the proof-mass will be acted on by a periodic Coriolis force and produce vibrations, so the MEMS gyroscope can sense an angular velocity input. The amplitude of the swing angle is proportional to the input angular velocity, while the frequency of the vibration corresponds to the spin frequency of the rotating aircraft. This vibration will be transformed into an alternating electrical signal via a capacitance detection circuit. The alternating electrical signal is called the output signal of the gyro. The specific working principle is shown in Figure 4.

As shown in Figure 4a, the coordinate system oxyz is fixed on the sensing element of the MEMS gyroscope. The axis oy is the output axis of the gyro (also called precession axis). The axis ox is the input axis, while the oz axis is called the drive axis. The axis oz coincides with the longitudinal axis of the rotating aircraft’s body. As the body of the aircraft spins around the axis oz with an angular velocity $\dot{\varphi }$, the sensing element will obtain the angular momentum. When the aircraft rotates around the axis ox at an angular rate Ω, it will force the sensing element to produce a precession motion around the same axis ox with the same angular rate Ω. Along the direction of the precession axis oy, the precession torque will act on the sensing element.

With the spin of the aircraft, both $\Omega_{x}$ and $\Omega_{y}$, which are on the quasi-body coordinate system $ox^{´}y^{´}z$, will be projected onto the sensing axis ox. Then the precession moment $M_{g}$ will act on the output axis oy, so the sensing element will produce a periodic vibration. An illustration of this movement can be found in Figure 4b.

Besides, the precession torque can be balanced by the moments, which are respectively the inertia moment, damping moment and elastic moment. The sensing element has an angular acceleration to produce the inertia moment. Because of the angular velocity $\dot{\alpha }$, a damping moment will exist between the sensing element and the thick film ceramic electrode. When the sensing element vibrates with angular velocity $\dot{\alpha }$ and has a swing angle of α, the elastic torsion beams will produce an elastic torsion moment.

Thus, the sensing element has a degree-of-freedom on the direction of axis oy, and can rotate around the axis oy. The sensing element cannot rotate around the axis ox because the two ends of the sensing element are fixed on the outer frame by the elastic torsion beams. However, the angular velocity input on ox axis can be sensed, and the axis ox is also called the sensing axis. Although the MEMS gyroscope does not have a drive structure like common gyroscopes, it has the feature that it senses the input angular velocity using the spin of the rotating carrier. In other words, the input of the MEMS gyroscope is the angular velocity on the ox axis, and the output is the precession movement around the oy axis.

## 3. The Motion Equation

This motion of the sensing element corresponds to the motion of a rigid body around a fixed point, so the motion equation can be derived by the Euler dynamics equation.

### 3.1. Building Reference Frame

**Figure 5:**
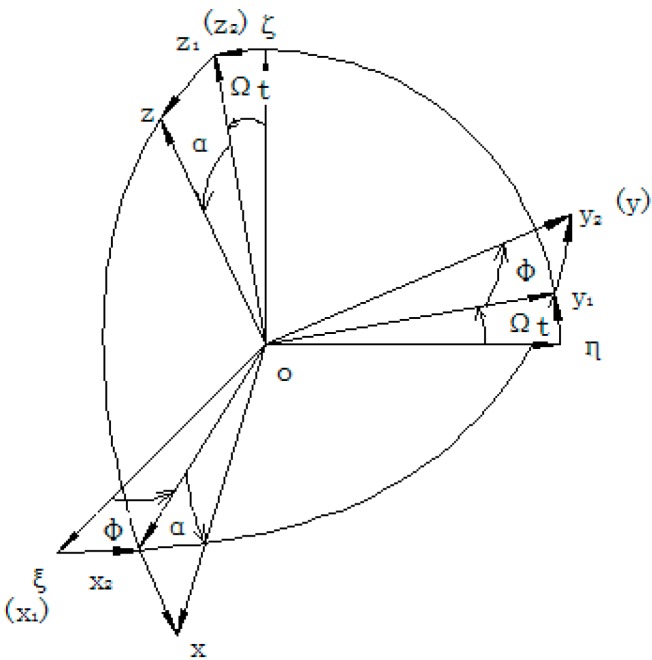
Transformation of the reference frame.

We choose four reference frames for studying the motion. As shown in Figure 5, oξης is an inertia reference frame, $ox_{1}y_{1}z_{1}$ is a transverse reference frame, $ox_{2}y_{2}z_{2}$ is a spin coordinate system, and oxyz is called a moving coordinate system, which is fixed on the sensing element. The origin of these reference frames is located on the mass center of the sensing element.

The transformation of reference frame is shown in Figure 5. First, inertia system oξης rotates to the transverse system $ox_{1}y_{1}z_{1}$ around oξ with an angular rate of Ω, and the angle is Ωt. Second, $ox_{1}y_{1}z_{1}$ rotates to $ox_{2}y_{2}z_{2}$ around $oz_{1}$ with an angular rate of $\dot{\varphi }$, and the angle is φ. Third, $ox_{2}y_{2}z_{2}$ rotates to move the coordinate system oxyz around $oy_{2}$ with an angular rate of $\dot{\alpha }$, and the angle is α. These angles are functions of time.

### 3.2. Deriving of Motion Equation

For the sensing element of the MEMS gyroscope, building the motion equation actually establishes an equation of the vibration around the precession axis oy. Firstly, from oξης to $ox_{1}y_{1}z_{1}$, the coordinate transformation is:

(1) $$\left[\begin{array}{c} x_{1}\\ y_{1}\\ z_{1} \end{array}\right]=\left[\begin{array}{ccc} 1 & 0 & 0\\ 0 & \cos\Omega t & \sin\Omega t\\ 0 & -\sin\Omega t & \cos\Omega t \end{array}\right]\left[\begin{array}{c} \xi \\ \eta \\ \varsigma \end{array}\right]$$

To define rotary matrix ${\text{A}}_{1}=\left[\begin{array}{ccc} 1 & 0 & 0\\ 0 & \cos\Omega t & \sin\Omega t\\ 0 & -\sin\Omega t & \cos\Omega t \end{array}\right]$, then:

(2) $$\left[\begin{array}{c} x_{1}\\ y_{1}\\ z_{1} \end{array}\right]={\text{A}}_{1}\left[\begin{array}{c} \xi \\ \eta \\ \varsigma \end{array}\right]$$

Then, from $ox_{1}y_{1}z_{1}$ to $ox_{2}y_{2}z_{2}$, the coordinate transformation is:

(3) $$\left[\begin{array}{c} x_{2}\\ y_{2}\\ z_{2} \end{array}\right]=\left[\begin{array}{ccc} \cos\varphi & \sin\varphi & 0\\ -\sin\varphi & \cos\varphi & 0\\ 0 & 0 & 1 \end{array}\right]\left[\begin{array}{c} x_{1}\\ y_{1}\\ z_{1} \end{array}\right]$$

To define rotary matrix ${\text{A}}_{2}=\left[\begin{array}{ccc} \cos\varphi & \sin\varphi & 0\\ -\sin\varphi & \cos\varphi & 0\\ 0 & 0 & 1 \end{array}\right]$, then:

(4) $$\left[\begin{array}{c} x_{2}\\ y_{2}\\ z_{2} \end{array}\right]={\text{A}}_{2}\left[\begin{array}{c} x_{1}\\ y_{1}\\ z_{1} \end{array}\right]$$

Submitting Equation (2) into Equation (4), gives:

(5) $$\left[\begin{array}{c} x_{2}\\ y_{2}\\ z_{2} \end{array}\right]={\text{A}}_{2}{\text{A}}_{1}\left[\begin{array}{c} \xi \\ \eta \\ \varsigma \end{array}\right]$$

Finally, from $ox_{2}y_{2}z_{2}$ to oxyz, the coordinate transformation is:

(6) $$\left[\begin{array}{c} x\\ y\\ z \end{array}\right]=\left[\begin{array}{ccc} \cos\alpha & 0 & -\sin\alpha \\ 0 & 1 & 0\\ \sin\alpha & 0 & \cos\alpha \end{array}\right]\left[\begin{array}{c} x_{2}\\ y_{2}\\ z_{2} \end{array}\right]$$

To define rotary matrix ${\text{A}}_{3}=\left[\begin{array}{ccc} \cos\alpha & 0 & -\sin\alpha \\ 0 & 1 & 0\\ \sin\alpha & 0 & \cos\alpha \end{array}\right]$, then:

(7) $$\left[\begin{array}{c} x\\ y\\ z \end{array}\right]={\text{A}}_{3}\left[\begin{array}{c} x_{2}\\ y_{2}\\ z_{2} \end{array}\right]$$

Substituting Equation (5) into Equation (7), gives:

(8) $$\left[\begin{array}{c} x\\ y\\ z \end{array}\right]={\text{A}}_{3}{\text{A}}_{2}{\text{A}}_{1}\left[\begin{array}{c} \xi \\ \eta \\ \varsigma \end{array}\right]$$

To define $\text{A}={\text{A}}_{3}{\text{A}}_{2}{\text{A}}_{1}$, this rotary transformation matrix is a function of time. The calculated expression is:

(9) $$\text{A}=\left[\begin{array}{ccc} \cos\varphi \cos\alpha & \cos\Omega t\cos\alpha \sin\varphi +\sin\Omega t\sin\alpha & \sin\Omega t\cos\alpha \sin\varphi -\cos\Omega t\sin\alpha \\ -\sin\varphi & \cos\varphi \cos\Omega t & \cos\varphi \sin\Omega t\\ \cos\varphi \sin\alpha & \cos\Omega t\sin\alpha \sin\varphi -\cos\alpha \sin\Omega t & \cos\Omega t\cos\alpha +\sin\alpha \sin\varphi \sin\Omega t \end{array}\right]$$

The rotary transformation matrix A is an orthogonal matrix that it can be expressed as ${\text{A}}^{-1}={\text{A}}^{T}$. Then, the inverse matrix of A can be expressed as:

(10) $${\text{A}}^{-1}=\left[\begin{array}{ccc} \cos\varphi \cos\alpha & -\sin\varphi & \cos\varphi \sin\alpha \\ \cos\Omega t\cos\alpha \sin\varphi +\sin\Omega t\sin\alpha & \cos\varphi \cos\Omega t & \cos\Omega t\sin\alpha \sin\varphi -\cos\alpha \sin\Omega t\\ \sin\Omega t\cos\alpha \sin\varphi -\cos\Omega t\sin\alpha & \cos\varphi \sin\Omega t & \cos\Omega t\cos\alpha +\sin\alpha \sin\varphi \sin\Omega t \end{array}\right]$$

In the inertial coordinate system, according to the moment of momentum theorem, we have:

(11) $$\frac{d\text{G}}{dt}=\text{M}$$

where G is the vector of the moment of momentum, M is the vector of the applied moment acting on the sensing element. In matrix form, Equation (11) can be expressed as:

(12) $$\frac{d}{dt}\left[\begin{array}{c} G_{\xi }\\ G_{\eta }\\ G_{\varsigma } \end{array}\right]=\left[\begin{array}{c} M_{\xi }\\ M_{\eta }\\ M_{\varsigma } \end{array}\right]$$

In the above equation, $\left[\begin{array}{c} G_{\xi }\\ G_{\eta }\\ G_{\varsigma } \end{array}\right]=\text{J}\left[\begin{array}{c} \omega_{\xi }\\ \omega_{\eta }\\ \omega_{\varsigma } \end{array}\right]$, J is the vector of the moment of inertia, $\left[\begin{array}{c} \omega_{\xi }\\ \omega_{\eta }\\ \omega_{\varsigma } \end{array}\right]$ is the vector of the angular velocity.

Additionally:

(13) $$\left[\begin{array}{c} G_{\xi }\\ G_{\eta }\\ G_{\varsigma } \end{array}\right]={\text{A}}^{-1}\left[\begin{array}{c} G_{x}\\ G_{y}\\ G_{z} \end{array}\right],\left[\begin{array}{c} M_{\xi }\\ M_{\eta }\\ M_{\varsigma } \end{array}\right]={\text{A}}^{-1}\left[\begin{array}{c} M_{x}\\ M_{y}\\ M_{z} \end{array}\right]$$

Plugging Equation (13) into Equation (12), we obtain:

(14) $$\frac{d}{dt}\left({\text{A}}^{-1}\left[\begin{array}{l} G_{x}\\ G_{y}\\ G_{z} \end{array}\right]\right)={\text{A}}^{-1}\left[\begin{array}{l} M_{x}\\ M_{y}\\ M_{z} \end{array}\right]$$

Expanding Equation (14) and multiplying by A the left side of Equation (14), we will have:

(15) $$\frac{d}{dt}\left[\begin{array}{l} G_{x}\\ G_{y}\\ G_{z} \end{array}\right]+\text{A}\frac{d{\text{A}}^{-1}}{dt}\left[\begin{array}{l} G_{x}\\ G_{y}\\ G_{z} \end{array}\right]=\left[\begin{array}{l} M_{x}\\ M_{y}\\ M_{z} \end{array}\right]$$

which gives:

(16) $$\text{A}\frac{d{\text{A}}^{-1}}{dt}=\left[\begin{array}{ccc} 0 & -\Omega \cos\varphi \sin\alpha -\dot{\varphi }\cos\alpha & \dot{\alpha }-\Omega \sin\varphi \\ \Omega \cos\varphi \sin\alpha +\dot{\varphi }\cos\alpha & 0 & \dot{\varphi }\sin\alpha -\Omega \cos\varphi \cos\alpha \\ \Omega \sin\varphi -\dot{\alpha } & \Omega \cos\varphi \cos\alpha -\dot{\varphi }\sin\alpha & 0 \end{array}\right]$$

Plugging this into Equation (15), gives:

(17) $$\frac{d}{dt}\left[\begin{array}{l} G_{x}\\ G_{y}\\ G_{z} \end{array}\right]+\left[\begin{array}{ccc} 0 & -\Omega \cos\varphi \sin\alpha -\dot{\varphi }\cos\alpha & \dot{\alpha }-\Omega \sin\varphi \\ \Omega \cos\varphi \sin\alpha +\dot{\varphi }\cos\alpha & 0 & \dot{\varphi }\sin\alpha -\Omega \cos\varphi \cos\alpha \\ \Omega \sin\varphi -\dot{\alpha } & \Omega \cos\varphi \cos\alpha -\dot{\varphi }\sin\alpha & 0 \end{array}\right]\left[\begin{array}{l} G_{x}\\ G_{y}\\ G_{z} \end{array}\right]=\left[\begin{array}{l} M_{x}\\ M_{y}\\ M_{z} \end{array}\right]$$

and because the moving coordinate system is the system of the inertial principal axis, we have:

(18) $$\text{J}=\left[\begin{array}{ccc} J_{x} & 0 & 0\\ 0 & J_{y} & 0\\ 0 & 0 & J_{z} \end{array}\right]$$

Then:

(19) $$\left[\begin{array}{l} G_{x}\\ G_{y}\\ G_{z} \end{array}\right]=\text{J}\left[\begin{array}{l} \omega_{x}\\ \omega_{y}\\ \omega_{z} \end{array}\right]=\left[\begin{array}{ccc} J_{x} & 0 & 0\\ 0 & J_{y} & 0\\ 0 & 0 & J_{z} \end{array}\right]\left[\begin{array}{l} \omega_{x}\\ \omega_{y}\\ \omega_{z} \end{array}\right]=\left[\begin{array}{l} J_{x}\omega_{x}\\ J_{y}\omega_{y}\\ J_{z}\omega_{z} \end{array}\right]$$

where in accordance with Figure 3 above, the projections on each axis of the moving coordinate system are:

(20) $$\left[\begin{array}{l} \omega_{x}\\ \omega_{y}\\ \omega_{z} \end{array}\right]=\left[\begin{array}{l} \Omega \cos\varphi \cos\alpha -\dot{\varphi }\sin\alpha \\ \dot{\alpha }-\Omega \sin\varphi \\ \dot{\varphi }\cos\alpha -\Omega \cos\varphi \sin\alpha \end{array}\right]$$

Plugging Equations (19) and (20) into Equation (17), three dynamic equations can be obtained. According to the principle of the gyro structure, the angular vibration of the proof-mass is around the output axis oy, so the dynamics equation of the MEMS gyroscope is:

(21) $$\begin{array}{l} \frac{1}{2}\left[\left(J_{x}+J_{z}\right)\Omega^{2}\cos^{2}\varphi +\left(J_{z}-J_{x}\right){\dot{\varphi }}^{2}\right]\sin2\alpha +\\ J_{x}\Omega \dot{\varphi }\cos\varphi \cos2\alpha -\left(J_{z}+J_{y}\right)\Omega \dot{\varphi }\cos\varphi +\\ J_{y}\ddot{\alpha }-J_{y}\frac{d\Omega }{dt}\sin\varphi =M_{y} \end{array}$$

where $M_{y}=-K_{T}\alpha -D\dot{\alpha }$. The term $K_{T}$ is the stiffness coefficient of the elastic torsion beams. D is damping coefficient.

In actual application, considering that the $\Omega \gg \dot{\varphi }$ and the $\dot{\alpha }\ll \dot{\varphi }$, we can ignore $\Omega^{2}$ and have sinα≈α,cos2α≈cosα≈1, $\frac{d\Omega }{dt}\approx 0$, whereby Equation (21) can be simplified as:

(22) $$J_{y}\ddot{\alpha }+D\dot{\alpha }+\left[\left(J_{z}-J_{x}\right){\dot{\varphi }}^{2}+K_{T}\right]\alpha =\left(J_{z}+J_{y}-J_{x}\right)\dot{\varphi }\Omega \cos\left(\dot{\varphi }t\right)$$

Equation (22) is the motion equation of the MEMS gyroscope. It is a second order ordinary differential equation.

### 3.3. The Solution of Motion Equation

Based on the sensing mechanism of the MEMS gyroscope and the Euler dynamics equation, we have obtained the motion equation of the sensing element. It is a differential equation of the swing angle α. If:

(23) $$\begin{array}{l} \omega_{n}=\sqrt{\frac{1}{J_{y}}\left[\left(J_{z}-J_{x}\right){\dot{\varphi }}^{2}+K_{T}\right]}\\ \zeta =\frac{D}{2\omega_{n}J_{y}}=\frac{D}{2\sqrt{J_{y}\left[\left(J_{z}-J_{x}\right){\dot{\varphi }}^{2}+K_{T}\right]}}\\ f_{0}=\frac{1}{J_{y}}\left(J_{z}+J_{y}-J_{x}\right)\Omega \dot{\varphi } \end{array}$$

Then, Equation (22) can be rearranged to:

(24) $$\ddot{\alpha }+2\zeta \omega_{n}\dot{\alpha }+{\omega_{n}}^{2}\alpha =f_{0}\cos\left(\dot{\varphi }t\right)$$

which when solved gives:

(25) $$\alpha =Ae^{-nt}\sin(\omega t+\delta )+B\cos(\dot{\varphi }t-\beta )$$

Equation (25) includes two parts. The first part is $Ae^{-nt}\sin(\omega t+\delta )$, which is known as a transient solution. Here, A and δ are the integral constants, which relate to the initial conditions. $\omega =\omega_{n}\sqrt{1-\zeta^{2}}$, $n=\zeta \omega_{n}$. n is called the damping factor, so the first part reveals that the transient solution is an attenuation of oscillation. Based on the dynamics parameters in Equation (23), the above transient process can be described using a step input. The decay time is about 13.5 ms, which exactly meets the requirements of engineering. It is said that the oscillation can rapidly decay to zero, so we should mainly consider the second part, which is called a steady state solution. The steady state solution is:

(26) $$\alpha =\frac{f_{0}}{\omega_{n}\sqrt{\left[1-{\left(\frac{\dot{\varphi }}{\omega_{n}}\right)}^{2}\right]+4{\left(\zeta \dot{\varphi }\right)}^{2}}}\cos\left(\dot{\varphi }t-\beta \right)$$

where, $\beta =\arctan\frac{2\zeta \frac{\dot{\varphi }}{\omega_{n}}}{1-{\left(\frac{\dot{\varphi }}{\omega_{n}}\right)}^{2}}$, it is a phase delay. By plugging Equation (23) into Equation (26), we can obtain the steady state solution, which is:

(27) $$\alpha =\frac{\left(J_{z}+J_{y}-J_{x}\right)\dot{\varphi }}{\sqrt{{\left[\left(J_{z}-J_{y}-J_{x}\right){\dot{\varphi }}^{2}+K_{T}\right]}^{2}+{\left(D\dot{\varphi }\right)}^{2}}}\Omega \cos\left(\dot{\varphi }t-\beta \right)$$

Ideally, the sensing element is fabricated to be symmetrical structure. Then the moment of inertia has relations of $J_{x}\approx J_{y}$ and $J_{z}\approx J_{y}+J_{x}$. Since it is desired to increase natural frequency $\omega_{n}=\sqrt{\frac{K_{T}}{J_{y}}}$, the stiffness coefficient $K_{T}$ is typically designed to be bigger, which is $K_{T}\gg D\dot{\varphi }$. Equation (27) can be simplified as $\alpha =\frac{\left(J_{z}+J_{y}-J_{x}\right)\dot{\varphi }}{K_{T}}\Omega \cos\left(\dot{\varphi }t-\beta \right)$. This also demonstrates that the sensitivity of the gyro depends on the spin frequency of the rotating aircraft, so the MEMS gyroscope is very suitable for attitude sensing and control of high rate rotating aircraft. According to Equation (27), the amplitude of the swing angle is:

(28) $$\alpha_{m}=\frac{\left(J_{z}+J_{y}-J_{x}\right)\dot{\varphi }}{\sqrt{{\left[\left(J_{z}-J_{y}-J_{x}\right){\dot{\varphi }}^{2}+K_{T}\right]}^{2}+{\left(D\dot{\varphi }\right)}^{2}}}\Omega$$

Equations (27) and (28) demonstrate that the output signal of the gyro is an amplitude-modulation signal (AM). The amplitude of the output signal is proportional to the input angular velocity, while the carrier frequency corresponds to the frequency of spin of the rotating aircraft.

## 4. Sensing Analysis

According to Equation (28), the sensitivity of the MEMS gyroscope can be calculated using the following formula:

(29) $$\frac{\partial \alpha_{m}}{\partial \Omega }=\frac{\left(J_{z}+J_{y}-J_{x}\right)\dot{\varphi }}{\sqrt{{\left[\left(J_{z}-J_{y}-J_{x}\right){\dot{\varphi }}^{2}+K_{T}\right]}^{2}+{\left(D\dot{\varphi }\right)}^{2}}}$$

Considering the structure of the sensing element, the moments of inertia approximately fulfill the formula $J_{z}-J_{y}-J_{x}\approx 0$, so Equation (29) can be simplified as:

(30) $$\frac{\partial \alpha_{m}}{\partial \Omega }=\frac{\left(J_{z}+J_{y}-J_{x}\right)\dot{\varphi }}{\sqrt{{K_{T}}^{2}+{\left(D\dot{\varphi }\right)}^{2}}}$$

Equation (30) shows that stiffness coefficient of the elastic torsion beams and damping coefficient are the keys to improving the sensitivity of the MEMS gyroscope. Considering the difficulty of MEMS fabrication process technology, in the structure design, we usually change the stiffness coefficient by adjusting the thickness of the elastic torsion beams to improve the gyro sensitivity. The sensitivity assuming that the spin frequency of the aircraft is 15 Hz and beam was etched to different thicknesses, is as shown in Table 1. With the increase of the thickness of the beam, the sensitivity decreases.

**Table 1:** The sensitivity of gyro for different beam thicknessess.

Thickness (µm)	Stiffness Coefficient (N·m)	Sensitivity(rad/(°/s))
25	4.443 × 10^−4^	1.822 × 10^−5^
48	3.145 × 10^−3^	1.808 × 10^−5^
75	1.200 × 10^−2^	1.643 × 10^−5^

**Figure 6:**
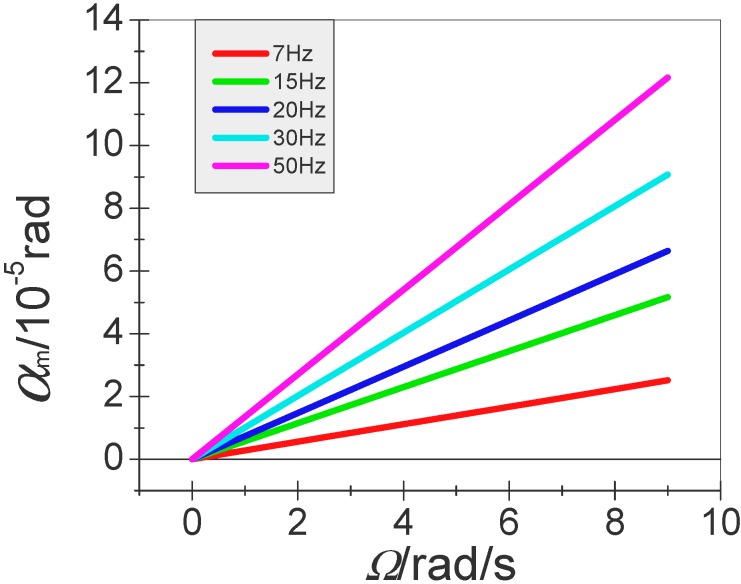
The relation curve between angular vibration amplitude and input angular velocity under different spin frequency.

In addition, under different spin frequency, the curve of the angular vibration amplitude of the input angular velocity is plotted in Figure 6.

Figure 6 shows that the vibration amplitude of the proof-mass is proportional to the input angular velocity when the spin frequency of the flying aircraft is under a certain condition.

## 5. Experimental Validation

For the steady state solution of the angular vibration equation, after the sensing signal is detected by the signal processing circuit, the output voltage signal of the gyroscope can be obtained. Equation (31) is the output voltage signal of the gyroscope, where K is the transmission coefficient of the signal processing circuit:

(31) $$u=K\frac{\left(J_{z}+J_{y}-J_{x}\right)\dot{\varphi }}{\sqrt{{\left[\left(J_{z}-J_{y}-J_{x}\right){\dot{\varphi }}^{2}+K_{T}\right]}^{2}+{\left(D\dot{\varphi }\right)}^{2}}}\Omega \cos\left(\dot{\varphi }t-\beta \right)$$

**Figure 7:**
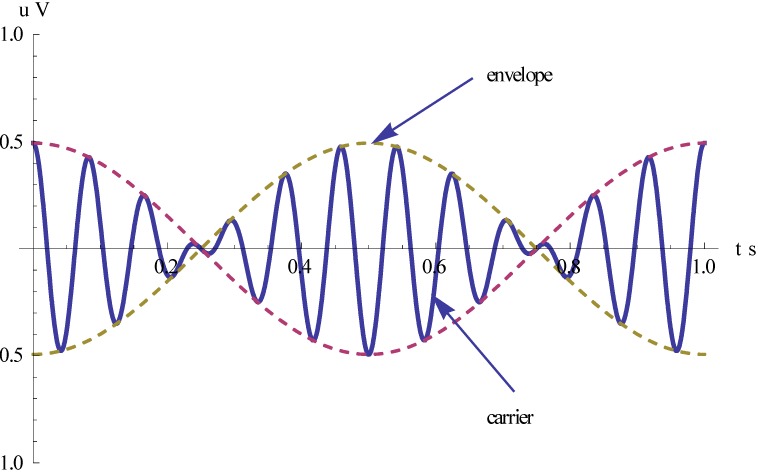
The output voltage signal of the gyro.

The non-driven sensing element shares the angular rotation of the aircraft. When the gyroscope is subjected to an angular rotation, a sinusoidal Coriolis force is induced. The rotation-induced Coriolis force causes an energy transfer to the sense-mode proportional to the angular velocity input. In other words, the gyro’s sensing signal is an AM signal. Its envelope is proportional to the input angular velocity, and the carrier frequency corresponds to the spin frequency as shown in Figure 7.

To validate that the above Equation (31) can correctly represent all sorts of attitude motions of the rotating aircraft, we will carry out an experimental validation under three common flight conditions of rotating aircraft.

**Figure 8:**
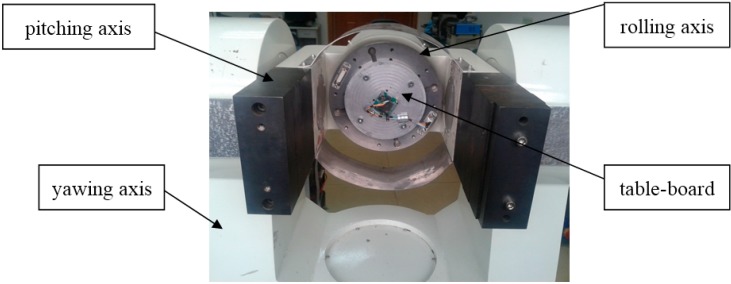
The experimental platform.

The experimental platform is a precision three axis rate table. It has three axis systems, which are respectively called the rolling axis, pitching axis and yawing axis. As shown in Figure 8, it uses a “U-U-T” structure. All three frameworks use mechanical bearings and form a shaft system. DC motors provide rotary torque. The optical encoder produces an angle signal, and the feedbacks of position and velocity are formed with this angle signal. Each framework is equipped with a conductive slip ring and dial.

The high precision rate table can simulate in the laboratory the attitude motion of a rotating aircraft. The inner framework is used to simulate the spin of the aircraft, and the middle framework and outer framework simulate the pitch and yaw, respectively. With the data acquisition card of the rate table, the output signal of the MEMS gyro can be obtained. Then these experimental data are compared with theoretical data to validate the correctness of the gyro signals. The main specifics of the rate table are listed in Table 2 below.

**Table 2:** The main specifics of the three axis rate table.

Angle	yaw	±360°	Position control accuracy	yaw	±0.001°	
	pitch	±360°		pitch	±0.001°	
	roll	±360°		roll	±0.3'	
Dynamic accuracy	yaw	±0.005°	Maximum angular acceleration	yaw	3000°/s^2^	
	pitch	±0.005°		pitch	4000°/s^2^	
Measured angle	roll	±0.2°		roll	4000°/s^2^	
Maximum angular velocity	yaw	±500°/s	Rate accuracy	yaw or pitch	0.001°/s~1°/s	1 × 10^−3^
	pitch	±500°/s			1°/s~10°/s	1 × 10^−4^
	roll	±50 r/s			10°/s	2 × 10^−5^
Minimum angular velocity	yaw	±0.001°/s		roll	1′/s~1°/s	2 × 10^−3^
	pitch	±0.001°/s			1°/s~10°/s	2 × 10^−4^
	roll	±1'/s			10°/s~2000°/s	2 × 10^−5^
					>2000°/s	2 × 10^−6^

The MEMS gyroscope is mounted on the table board, and rolling axis is set to 12 Hz to simulate the spin frequency of a rotating aircraft.

### 5.1. Input Constant Angular Velocity

When the flight carrier is subjected to a constant pitching or yawing angular velocity, the output signal is a sine-wave signal based on the expression given by Equation (31). In the tests, we enter an angular rate of 180°/s on the pitching axis. Then the output voltage is calibrated to RMS = 2 V.

**Figure 9:**
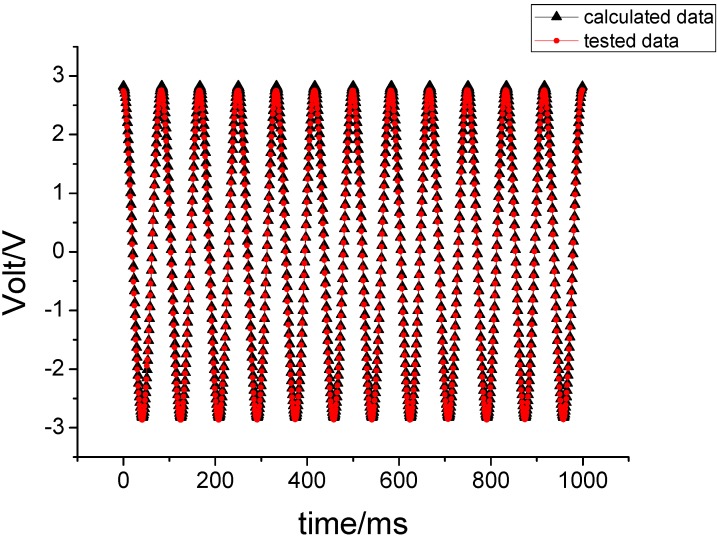
The experimental results when 180°/s is entered on the pitching axis.

Under the above experimental conditions, the output signal of the gyro is sampled with a sampling frequency of 1 KHz. The sampling data were compared with the theoretical values, as shown in Figure 9.

**Figure 10:**
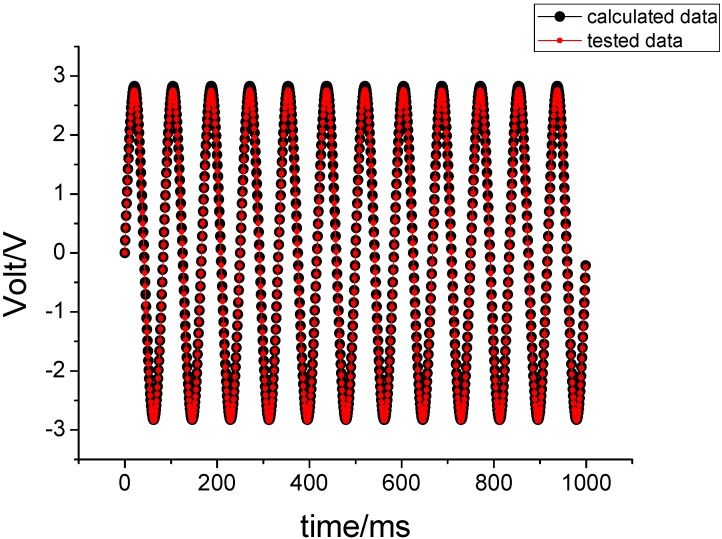
The experimental results when 180°/s is entered on the yawing axis.

Under unchanged conditions, 180°/s is entered for the yawing axis and the corresponding experimental results are shown in Figure 10.

Figure 9 shows that the correlation coefficient between tested value and theoretical value is 0.9969, while the correlation coefficient is 1.0000 in Figure 10. These results demonstrate that both the output signal of the gyroscope and the calculated value can coincide each other. This also demonstrates that the derived motion equation is correct under the input constant angular velocity.

### 5.2. Input Angular Vibration

When inputting the angular vibration and assuming that vibration amplitude is A and the vibration frequency is f, the angular displacement is:

(32) β=Asin2πft

To derivate Equation (32) for time, the input angular velocity is given by:

(33) $$\Omega =\frac{d\beta }{dt}=2\pi fA\cos2\pi ft$$

Now, the output signal of gyro is an amplitude modulation signal of the double sideband of the suppressed carrier. In order to perform the experimental verification, we enter the angular vibration on the pitching axis of rate table, of which amplitude and frequency are respectively 5° and 1 Hz.

**Figure 11:**
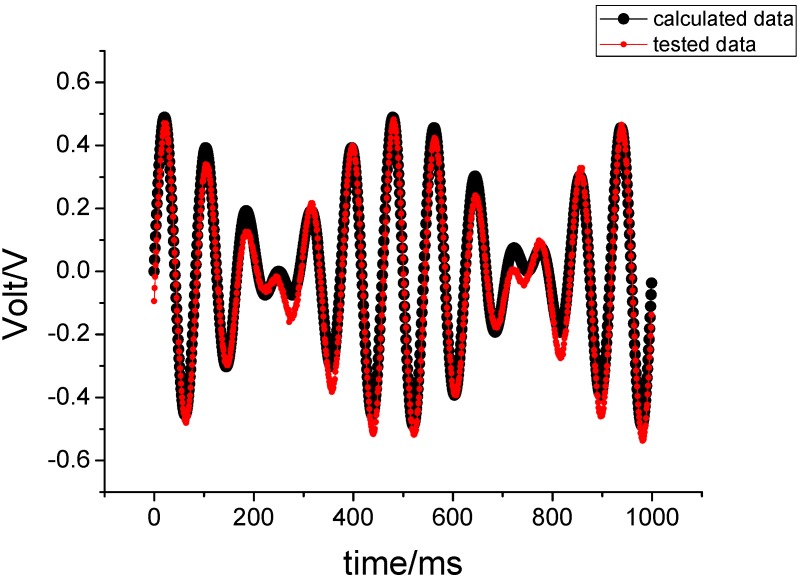
The experimental results under input angular vibration.

Under the above conditions, the sampled data were compared with the theoretical values, as shown in Figure 11.

Figure 11 shows that the correlation coefficient R equals 0.9872, so the experimental test and theoretical calculation coincide under input angular vibration. The simulation demonstrates that the gyro can correctly sense the rate of input angular vibration when a rotating aircraft is subjected to an angular vibration on the pitch axis or yaw axis. The envelope of the output signal is proportional to the rate of the input angular vibration.

### 5.3. Input Circular Vibration

When the flight carrier produces a circular vibration, in sensing plane, the angular vibrations would respectively be:

(34) $$\begin{array}{l} \beta_{x}=A\sin\left(2\pi ft+\theta_{x}\right)\\ \beta_{y}=A\sin\left(2\pi ft+\theta_{y}\right) \end{array}$$

To derivate Equation (34) for time, the input angular velocities are:

(35) $$\begin{array}{l} \Omega_{x}=\frac{d\beta_{x}}{dt}=2\pi fA\cos\left(2\pi ft+\theta_{x}\right)\\ \Omega_{y}=\frac{d\beta_{y}}{dt}=2\pi fA\cos\left(2\pi ft+\theta_{y}\right) \end{array}$$

**Figure 12:**
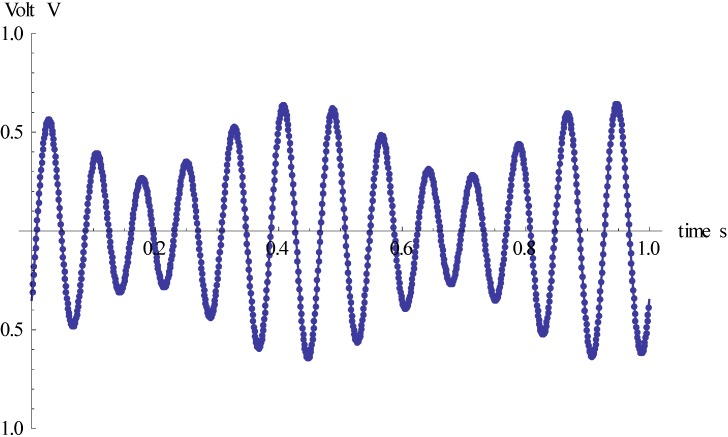
The output signal waveform of gyro under input circular vibration.

Thus, under circular vibration conditions, the output signal of the gyro is a resultant and complex amplitude modulation signal. When the phase difference is 135°, the corresponding output signal waveform is shown in Figure 12.

**Figure 13:**
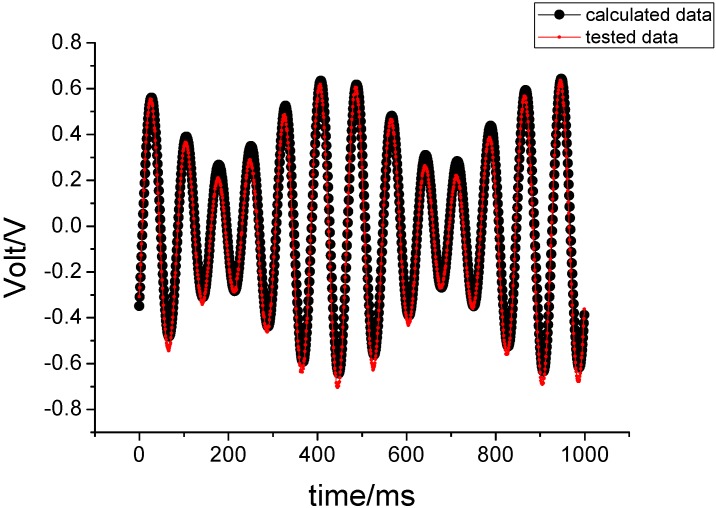
The experimental results under input circular vibration.

In the test, in order to simulate the circular vibration, we enter an angular vibration of amplitude 5° and frequency 1 Hz on the pitching axis and yawing axis, respectively. The phase difference of the vibration is 135°. Figure 13 compares the results of the sampling data and theoretical calculation data. Their comparison has a correlation coefficient of 0.9842, and they are also consistent in this case.

The simulation results in Figure 13 demonstrate that the gyro can correctly sense the resultant angular velocity of pitch and yaw, even though the rotating aircraft is a very complex condition where it is simultaneously subjected to an angular vibration on the pitch axis or yaw axis. The envelope of the output signal is proportional to the value of the resultant angular velocity.

To summarize the above three cases, the design of the MEMS gyro is rational and the derived motion equation is also correct. In applications, the MEMS gyroscope is installed on a rotating aircraft. It uses the spin of the rotating aircraft as a driving force. When the carrier produces a transverse rotation, the MEMS gyroscope can sense the input angular velocity. Based on the Euler dynamics equation, the motion equation of the gyro has been derived. Its solution reveals that the vibration amplitude of the proof-mass is proportional to the input angular velocity, and the vibration frequency responds to the spin frequency of the aircraft, so the proposed MEMS gyroscope is very suitable for damping of a single-channel control system of a rotating aircraft.

Using a rate table, experimental tests have been done for three cases of flight attitude. The results have shown that the design is rational and the obtained motion equations are actually correct. However, the sensitivity of gyroscope would be affected by the spin frequency of the rotating aircraft. With the increase of the spin frequency, the sensitivity increases. This change will cause instability of the scale factor of gyroscope. For a single channel control system, this change is acceptable. In a multi-channel control system application, in order to guarantee the measuring accuracy of the MEMS gyroscope, we need to compensate this influence.

**Figure 14:**
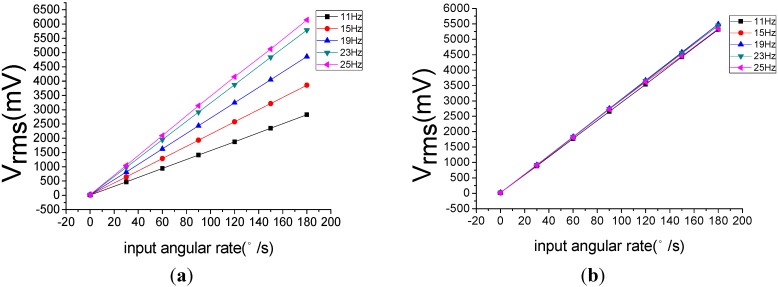
(**a**) The change before compensation; (**b**) the change after compensation.

Figure 14is a comparison of the scale factor before and after the compensation. Figure 14a shows that the scale factor will change with the change of spin frequency, while the Figure 14b demonstrates that the change of the scale factor has been effectively restrained.

## 6. Conclusions

The MEMS gyroscope which has been introduced in this paper, is a novel MEMS gyroscope that differs from the common MEMS gyroscope. It utilizes the spin of a rotating aircraft to attain angular momentum, as it has no complex drive structure itself like the common gyroscope, and does not need to achieve resonance to attain maximum response gain and sensitivity, so its structure is simple and the fabrication process is also easy. The non-driven MEMS gyroscope is installed on a rotating aircraft. When the flight carrier is subjected to a rotation, it can sense the angular velocity input and the carrier frequency of the output signal can automatically change with the change of spin frequency of the rotating aircraft, making it very suitable for the damping of a single-channel control system of a rotating aircraft. In the paper, its sensing mechanism has been analyzed in detail. It is found that the motion of the proof-mass is a precession motion. In the design of the force re-balance loop, this precession torque is balanced by the inertia torque, elastic torque and damping torque. The motion equation has been derived based on the Euler dynamics equation of a rigid body around a fixed point. Meanwhile, we have solved the motion equation and analyzed its solution. Finally, based on a precision three axis rate table, experimental tests have been done to validate the design and derived motion equation. The three correlation coefficients between the test data and the calculated values are 0.9969, 0.9872 and 0.9842, respectively. These experimental results have demonstrated that the design is rational and derived motion equation is actually correct.
